# Supplementary figures and images for: Development and validation of an assay for detection of Japanese encephalitis virus specific antibody responses
Source: PLoS One. 2020 Oct 28;15(10):e0238609. doi: 10.1371/journal.pone.0238609 (PMC7592747; doi:10.1371/journal.pone.0238609)

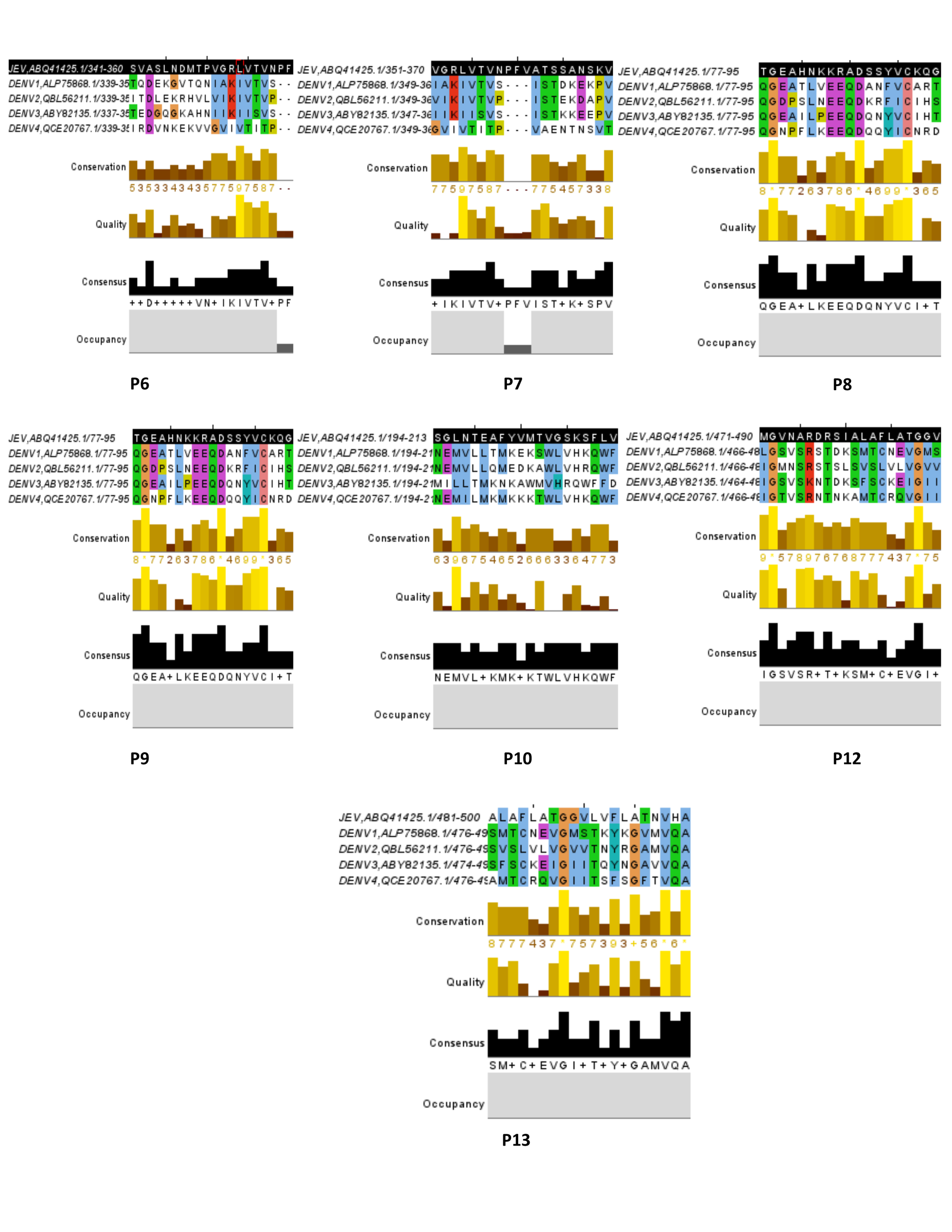

Supplement: S1 Fig — (TIF) [file pone.0238609.s001.tif]

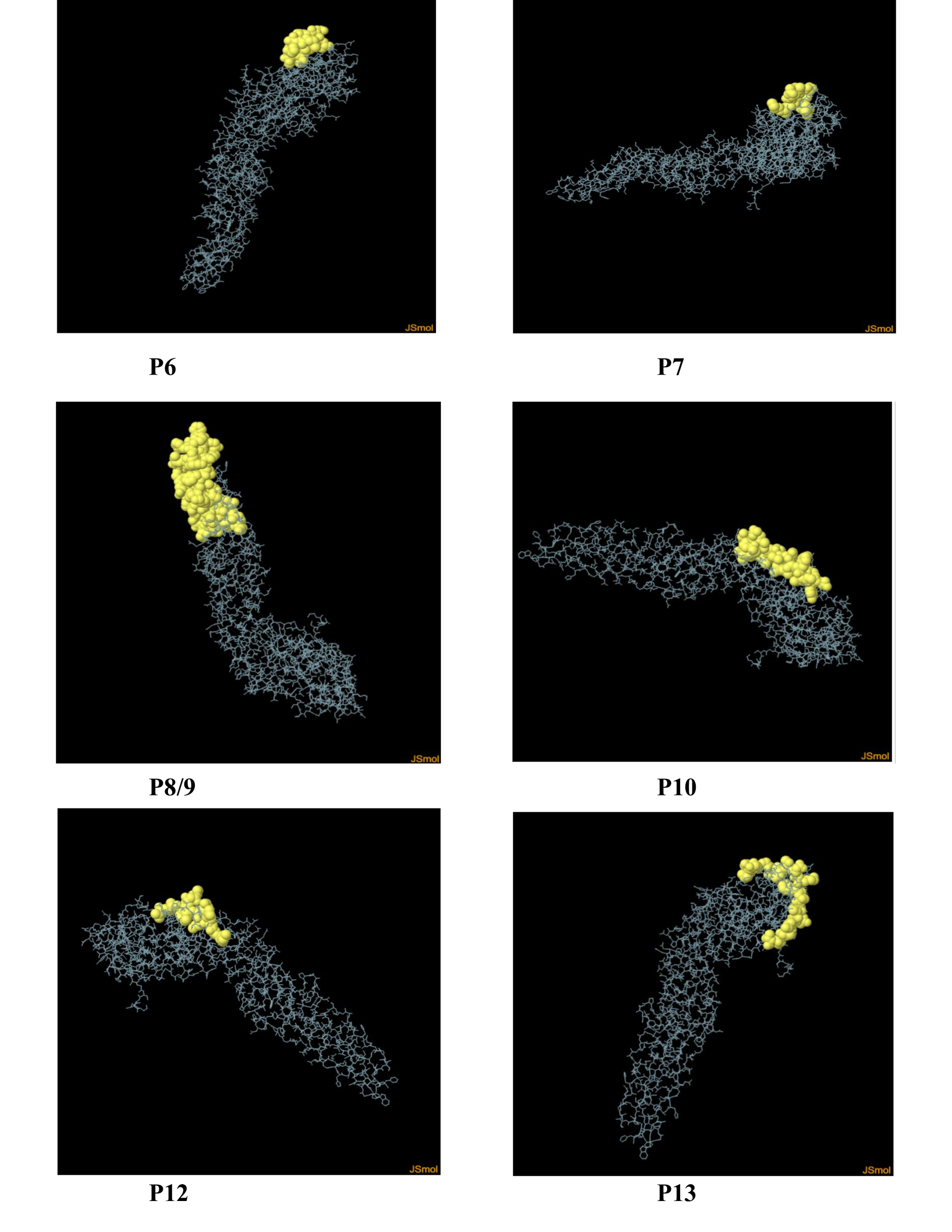

Supplement: S2 Fig — (TIF) [file pone.0238609.s002.tif]
