# Supplementary material for: Development and validation of an assay for detection of Japanese encephalitis virus specific antibody responses
Source: PLoS One. 2020 Oct 28;15(10):e0238609. doi: 10.1371/journal.pone.0238609 (PMC7592747; doi:10.1371/journal.pone.0238609)
Supplement: S1 Table — (DOCX) [file pone.0238609.s003.docx]

**S1 Table. The homology of JEV-specific commercially synthesized peptides with four dengue serotypes, WNV, YFV and Zika virus**

| No | Peptide sequence | Protein | Peptide ID | DENV1% | DENV2% | DENV3% | DENV4% | WNV% | YFV% | Zika % |
| --- | --- | --- | --- | --- | --- | --- | --- | --- | --- | --- |
| 1 | ^20^GLPRVFPLVGVKRVVMSLLDG^39^ | Capsid | P1 | 30 | 25 | 30 | 30 | 55 | 50 | 40 |
| 2 | ^155^YSAQVGASQAAKFTVTPNAP^174^ | Envelope | P2 | 30 | 15 | 35 | 15 | 55 | 20 | 25 |
| 3 | ^149^SENHGNYSAQVGASQAAKFT^168^ | Envelope | P3 | 25 | 25 | 25 | 25 | 55 | 35 | 25 |
| 4 | ^331^SDGPCKIPIVSVASLNDMTP^350^ | Envelope | P5 | 35 | 30 | 30 | 25 | 75 | 35 | 35 |
| 5 | ^341^SVASLNDMTPVGRLVTVNPF^360^ | Envelope | P6 | 35 | 35 | 25 | 25 | 90 | 35 | 40 |
| 6 | ^351^VGRLVTVNPFVATSSANSKV^370^ | Envelope | P7 | 30 | 35 | 35 | 30 | 75 | 30 | 40 |
| 7 | ^53^LAEVRSYCYHASVTDISTVA^72^ | Envelope | P8 | 20 | 30 | 25 | 35 | 70 | 30 | 45 |
| 8 | ^77^TGEAHNKKRADSSYVCKQG^95^ | Envelope | P9 | 30 | 25 | 35 | 25 | 60 | 30 | 40 |
| 9 | ^194^SGLNTEAFYVMTVGSKSFLV^213^ | Envelope | P10 | 25 | 20 | 25 | 25 | 65 | 30 | 35 |
| 10 | ^261^GLHQALAGAIVVEYSSSVKL^280^ | Envelope | P11 | 30 | 35 | 25 | 30 | 75 | 35 | 30 |
| 11 | ^471^MGVNARDRSIALAFLATGGV^490^ | Envelope | P12 | 30 | 25 | 20 | 35 | 80 | 15 | 45 |
| 12 | ^481^ALAFLATGGVLVFLATNVHA^500^ | Envelope | P13 | 25 | 25 | 25 | 35 | 70 | 20 | 50 |
| 13 | ^121^LQIGVHGILNAAAIAWMIVR^140^ | NS2A | P14 | 15 | 15 | 15 | 20 | 40 | 25 | 25 |
| 14 | ^108^NESSIMWLASLAIVTACAG^126^ | Capsid | P16 | 15 | 15 | 15 | 20 | 20 | 30 | 25 |
| 15 | ^73^SSQAGSLFVLPRGVPFTDLD^92^ | NS4B | P18 | 30 | 30 | 35 | 30 | 50 | 25 | 40 |
| 16 | ^11^ADLKSMFAGKTQASGLTGLP^33^ | NS4B | P19 | 20 | 20 | 20 | 20 | 40 | 25 | 30 |
| 17 | ^21^TQASGLTGLPSMALDLRPAT^40^ | NS4B | P20 | 20 | 25 | 20 | 20 | 50 | 30 | 35 |
| 18 | ^270^GEVHSNQEKIKKRIQKLKEE^289^ | NS5 | P30 | 30 | 35 | 30 | 25 | 40 | 25 | 20 |
| 19 | ^633^GPQHLEQLPRKNKIAV^648^ | NS5 | P31 | 30 | 25 | 25 | 20 | 40 | 20 | 30 |
| 20 | ^100^KQNKRGGNEGSIMWLACLAV^119^ | Capsid | P32 | 10 | 15 | 10 | 25 | 40 | 25 | 25 |
| 21 | ^105^QITLTTFLTAMVLATLHYGY^124^ | NS4B | P33 | 25 | 25 | 25 | 30 | 60 | 25 | 35 |
| 22 | ^111^AAFFQLASADLQIGVHGILN^130^ | NS2A | P34 | 20 | 10 | 10 | 20 | 40 | 25 | 25 |
