# Supplementary material for: Development and validation of an assay for detection of Japanese encephalitis virus specific antibody responses
Source: PLoS One. 2020 Oct 28;15(10):e0238609. doi: 10.1371/journal.pone.0238609 (PMC7592747; doi:10.1371/journal.pone.0238609)
Supplement: S2 Table — (DOCX) [file pone.0238609.s004.docx]

**S2 Table. JEV specific 22 peptides antibody responses for individuals who have had varied JEV and DENV seropositivity**

| Peptide ID | Protein | Number of individuals who responded | | | Median (IQR) OD | | |
| --- | --- | --- | --- | --- | --- | --- | --- |
|  |  | JEV^-^DENV^+^ (n=30) | JEV^+^DENV^-^ (n=30) | JEV^+^DENV^+^ (n=30) | JEV^-^DENV^+^ | JEV^+^DENV^-^ | JEV^+^DENV^+^ |
| P1 | Capsid | 1 | 16 | 18 | 0.277 (0.247 to 0.310) | 0.430 (0.342 to 0.769) | 0.450 (0.329 to 0.799) |
| P2 | Envelope | 2 | 15 | 15 | 0.275 (0.230 to 0.320) | 0.430 (0.332 to 0.831) | 0.417 (0.308 to 0.909) |
| P3 | Envelope | 0 | 17 | 17 | 0.270 (0.234 to 0.290) | 0.385 (0.306 to 0.932) | 0.456 (0.303 to 0.845) |
| P5 | Envelope | 1 | 23 | 22 | 0.272 (0.254 to 0.338) | 0.820 (0.446 to 1.464) | 0.863 (0.372 to 1.328) |
| P6 | Envelope | 0 | 29 | 28 | 0.274 (0.252 to 0.307) | 1.254 (0.776 to 1.827) | 1.231 (0.950 to 1.733) |
| P7 | Envelope | 0 | 30 | 30 | 0.269 (0.249 to 0.316) | 1.515 (1.001 to 2.388) | 1.646 (1.070 to 2.482) |
| P8 | Envelope | 1 | 30 | 30 | 0.280 (0.259 to 0.322) | 1.673 (1.334 to 2.055) | 1.661 (1.085 to 2.049) |
| P9 | Envelope | 0 | 30 | 30 | 0.281 (0.268 to 0.311) | 1.807 (1.540 to 2.445) | 1.882 (1.630 to 2.266) |
| P10 | Envelope | 0 | 28 | 30 | 0.279 (0.268 to 0.310) | 1.474 (1.284 to 1.991) | 1.521 (1.089 to 1.883) |
| P11 | Envelope | 0 | 24 | 28 | 0.262 (0.206 to 0.279) | 1.099 (0.406 to 1.499) | 1.196 (0.696 to 1.476) |
| P12 | Envelope | 0 | 30 | 29 | 0.280 (0.262 to 0.303) | 1.509 (1.065 to 1.976) | 1.509 (1.135 to 1.720) |
| P13 | Envelope | 1 | 30 | 30 | 0.271 (0.258 to 0.314) | 1.572 (1.107 to 1.955) | 1.564 (1.281 to 1.944) |
| P14 | NS2A | 0 | 2 | 1 | 0.271 (0.256 to 0.305) | 0.282 (0.263 to 0.289) | 0.255 (0.233 to 0.267) |
| P16 | Capsid | 0 | 22 | 24 | 0.296 (0.265 to 0.320) | 0.829 (0.343 to 1.470) | 0.897 (0.339 to 1.481) |
| P18 | NS4B | 0 | 4 | 2 | 0.277 (0.261 to 0.307) | 0.286 (0.268 to 0.316) | 0.286 (0.262 to 0.313) |
| P19 | NS4B | 0 | 3 | 1 | 0.266 (0.247 to 0.300) | 0.294 (0.270 to 0.312) | 0.296 (0.270 to 0.318) |
| P20 | NS4B | 0 | 2 | 4 | 0.268 (0.252 to 0.308) | 0.292 (0.265 to 0.316) | 0.278 (0.261 to 0.318) |
| P30 | NS5 | 1 | 5 | 6 | 0.270 (0.246 to 0.307) | 0.296 (0.271 to 0.328) | 0.281 (0.264 to 0.337) |
| P31 | NS5 | 2 | 5 | 4 | 0.278 (0.254 to 0.305) | 0.281 (0.259 to 0.332) | 0.277 (0.260 to 0.315) |
| P32 | Capsid | 0 | 24 | 26 | 0.261 (0.248 to 0.310) | 1.135 (0.687 to 1.570) | 1.191 (0.772 to 1.824) |
| P33 | NS4B | 2 | 4 | 3 | 0.286 (0.265 to 0.321) | 0.292 (0.263 to 0.333) | 0.277 (0.258 to 0.293) |
| P34 | NS2A | 0 | 3 | 4 | 0.266 (0.242 to 0.299) | 0.279 (0.257 to 0.307) | 0.267 (0.245 to 0.285) |
