# Supplementary material for: Development and validation of an assay for detection of Japanese encephalitis virus specific antibody responses
Source: PLoS One. 2020 Oct 28;15(10):e0238609. doi: 10.1371/journal.pone.0238609 (PMC7592747; doi:10.1371/journal.pone.0238609)
Supplement: S3 Table — (DOCX) [file pone.0238609.s005.docx]

**S3 Table: Epitope prediction by four B cell linear epitope prediction servers (Bepipred, Elipro, BCEpred, and ABCpred)**

| No | Peptide sequence | Protein | Peptide ID | Bepipred^*^ | Elipro^#^ | BCEpred^@^ | ABCpred^$^ |
| --- | --- | --- | --- | --- | --- | --- | --- |
| 1 | ^341^SVASLNDMTPVGRLVTVNPF^360^ | Envelope | P6 | Y | Y | Y | Y |
| 2 | ^351^VGRLVTVNPFVATSSANSKV^370^ | Envelope | P7 | Y | Y | Y | Y |
| 3 | ^53^LAEVRSYCYHASVTDISTVA^72^ | Envelope | P8 | Y | Y | Y | Y |
| 4 | ^77^TGEAHNKKRADSSYVCKQG^95^ | Envelope | P9 | Y | Y | Y | Y |
| 5 | ^194^SGLNTEAFYVMTVGSKSFLV^213^ | Envelope | P10 | Y | Y | Y | Y |
| 6 | ^471^MGVNARDRSIALAFLATGGV^490^ | Envelope | P12 | N | N | Y | Y |
| 7 | ^481^ALAFLATGGVLVFLATNVHA^500^ | Envelope | P13 | N | N | Y | Y |

Y- Predicted N- not predicted

* www.iedb.org

# [www.iedb.org](http://www.iedb.org)

@ crdd.osdd.net, Saha.S and Raghava G.P.S. BcePred:Prediction of Continuous B-Cell Epitopes in Antigenic Sequences Using Physico-chemical Properties. In G.Nicosia, V.Cutello, P.J. Bentley and J.Timis (Eds.) ICARIS 2004, LNCS 3239, 197-204, Springer,2004.

$ crdd.osdd.net, Saha, S and Raghava G.P.S. (2006) Prediction of Continuous B-cell Epitopes in an Antigen Using Recurrent Neural Network. Proteins,65(1),40-48 PMID: 16894596
