# Supplementary material for: Development and validation of an assay for detection of Japanese encephalitis virus specific antibody responses
Source: PLoS One. 2020 Oct 28;15(10):e0238609. doi: 10.1371/journal.pone.0238609 (PMC7592747; doi:10.1371/journal.pone.0238609)
Supplement: S4 Table — (DOCX) [file pone.0238609.s006.docx]

**S4 Table. PanBio units of JEV^-^DENV^+^ thirty individuals**

| Sample ID | PanBio units |
| --- | --- |
| 1 | 1.462 |
| 2 | 1.701 |
| 3 | 1.645 |
| 4 | 1.871 |
| 5 | 1.827 |
| 6 | 1.891 |
| 7 | 1.945 |
| 8 | 1.770 |
| 9 | 1.691 |
| 10 | 2.047 |
| 11 | 1.762 |
| 12 | 1.700 |
| 13 | 1.642 |
| 14 | 1.765 |
| 15 | 1.728 |
| 16 | 1.653 |
| 17 | 1.749 |
| 18 | 1.728 |
| **19** | **1.619** |
| 20 | 1.682 |
| 21 | 1.537 |
| **22** | **1.779** |
| 23 | 1.858 |
| 24 | 1.704 |
| 25 | 2.033 |
| **26** | **1.628** |
| 27 | 1.803 |
| 28 | 1.783 |
| 29 | 1.800 |
| 30 | 1.804 |

*JEV^-^DENV^+^ individuals who gave positive responses to 7 peptide pool are made bold
