# Supplementary material for: Development and validation of an assay for detection of Japanese encephalitis virus specific antibody responses
Source: PLoS One. 2020 Oct 28;15(10):e0238609. doi: 10.1371/journal.pone.0238609 (PMC7592747; doi:10.1371/journal.pone.0238609)
Supplement: S1 File — (DOCX) [file pone.0238609.s007.docx]

**Classification of past dengue disease severity in our cohort of healthy individuals**

An interviewer administered questionnaire was used to record demographic details, details of JE immunization status, and whether the participants required hospitalization for a febrile illness in the past in the 1689 healthy individuals who were recruited to this study [1, 2]. In those who had been hospitalized due to a febrile illness, further details were obtained in order to verify if they were hospitalized due to a dengue infection and if so the severity of illness. All those who reported to have past dengue infection and were thus hospitalized, were laboratory confirmed by either being NS1 positive and or DENV IgM/ IgG positive at the time of infection. These data were obtained from the diagnosis card given by the hospitals on discharge. Those who had never been hospitalized in the past were considered to have had an inapparent dengue infection (NSD) and those who had been hospitalized due to a dengue infection were classified has having DF and DHF based on the WHO 2011 guidelines [3]. 175/1689 individuals, who were found to have DHF (SD) in the past, we included in the comparison of JEV peptide specific antibody responses with 175 individuals who had past inapparent dengue (NSD).

**A novel T cell-based assay to detect past infecting DENV serotype**

This novel T cell-based assay is carried out using a panel of DENV serotype specific peptides from highly conserved regions of the DENV, in a cultured ELISpot assay [2, 4, 5]. These peptides did not share any homology with other flaviviruses [4, 5]. PBMC obtained from DENV seropositive individuals from the community each donor was incubated with a pool of peptides consisting of all the 17 serotype-specific peptides. There were four peptides specific to DENV-1, five specific to DENV-2, four specific for DENV-3 and four specific for DENV-4. T cell lines were tested individually after 10 days culture for responses to the 17 serotype-specific peptides [4, 5]. All peptides that induced an IFN-γ response of more than mean+3 standard deviations of the control wells were considered positive. From this cohort of individuals in this study [2], we used sera of individuals who responded to only one DENV serotype and were thus considered to be only immune to that particular DENV serotype. In this large cohort study, none of the DENV seronegative individuals responded to any of these peptides and those who had a primary dengue infection during the study period were found to only respond to peptides of one DENV serotype [2].

**Reference**

1. Jeewandara, C. *et al.* Change in Dengue and Japanese Encephalitis Seroprevalence Rates in Sri Lanka. *PloS one* **10**, e0144799 (2015).
2. Jeewandara, C. *et al.* Functionality of dengue virus specific memory T cell responses in individuals who were hospitalized or who had mild or subclinical dengue infection. *PLoS neglected tropical diseases* **9**, e0003673 (2015).
3. WHO (ed). *Comprehensive guidelines for prevention and control of dengue fever and dengue haemorrhagic fever*. World Health Organization: SEARO, New Delhi, India, 2011.
4. Malavige, G.N. *et al.* Identification of serotype-specific T cell responses to highly conserved regions of the dengue viruses. *Clinical and experimental immunology* **168**, 215-223 (2012).
5. Jeewandara, C., Ogg, G.S. & Malavige, G.N. Cultured ELISpot Assay to Investigate Dengue Virus Specific T-Cell Responses. *Methods Mol Biol* **1808**, 165-171 (2018).
